# Mapping the immune landscape in small cell lung cancer by analysing expression of immuno-modulators in tissue biopsies and paired blood samples

**DOI:** 10.1038/s41598-023-30841-3

**Published:** 2023-03-06

**Authors:** Rimlee Dutta, Amber Rathor, Hanuman Prasad Sharma, Hem Chandra Pandey, Prabhat Singh Malik, Anant Mohan, Aruna Nambirajan, Rajeev Kumar, Deepali Jain

**Affiliations:** 1 Department of Pathology, All India Institute of Medical Sciences, Ansari Nagar, New Delhi, 110029 India; 2 Bioanalytics Facility, Centralized Core Research Facility, All India Institute of Medical Sciences, New Delhi, India; 3 Department of Transfusion Medicine, All India Institute of Medical Sciences, New Delhi, India; 4 Department of Medical Oncology, Dr B.R.A. Institute Rotary Cancer Hospital, All India Institute of Medical Sciences, New Delhi, India; 5 Department of Pulmonary Medicine, All India Institute of Medical Sciences, New Delhi, India; 6 Delhi Cancer Registry, Dr B.R.A. Institute Rotary Cancer Hospital, All India Institute of Medical Sciences, New Delhi, India

**Keywords:** Cancer, Biomarkers, Diseases, Medical research, Oncology

## Abstract

Small cell lung carcinomas (SCLC) are aggressive tumors with high propensity to metastasize. Recent NCCN guidelines have incorporated immunotherapy in extensive stage SCLC. Limited benefit in few patients compounded by side effects of unwonted immune-checkpoint-inhibitor (ICPI) usage necessitates identification of potential biomarkers predicting response to ICPIs. Attempting this, we analysed expression of various immunoregulatory molecules in tissue biopsies and paired blood samples of SCLC patients. In 40 cases, immunohistochemistry for expression of immune inhibitory receptors CTLA-4, PD-L1 and IDO1 was performed. Matched blood samples were quantified for IFN-γ, IL-2, TNF-α and sCTLA-4 levels using immunoassay and additionally for IDO1 activity (Kynurenine/Tryptophan ratio) using LC–MS. Immunopositivity for PD-L1, IDO1 and CTLA-4 was identified in 9.3%, 6.2% and 71.8% cases, respectively. Concentration of serum IFN-γ (p-value < 0.001), TNF-α (p-value = 0.025) and s-CTLA4 (p-value = 0.08) were higher in SCLC patients while IL-2 was lower (p-value = 0.003) as compared to healthy controls. IDO1 activity was significantly elevated in SCLC cohort (p-value = 0.007). We proffer that SCLC patients show immune suppressive milieu in their peripheral circulation. Analysis of CTLA4 immunohistochemical expression along with s-CTLA4 levels appears prospective as biomarkers for predicting responsiveness to ICPIs. Additionally, evaluation of IDO1 appears cogent both as prognostic marker and potential therapeutic target as well.

## Introduction

Accounting for about 15% of lung cancers globally, small cell lung cancer (SCLC) is a belligerent tumor subtype with approximately 70% of cases presenting with extensive disease at diagnosis^1^. Conventional tumor staging divides SCLC into limited stage (LS-SCLC) and extensive stage (ES-SCLC) disease. In most patients, for many years, platinum based chemotherapy has formed the standard first line of therapy for ES-SCLC with overall response rate of 40–70%, however responses to second line chemotherapy has been not so sanguine^2^. Despite therapy, SCLC are aggressive with a high propensity to metastasize or recur. This has led to exploration of newer modalities of therapy, with added emphasis on targeted therapy and immunotherapy. The relevance of immunogenicity of SCLC, has been evinced by recent studies, based on tumor infiltration by numerous T-lymphocytes along with occurrence of frequent paraneoplastic syndromes owing to autoantibody production and the close relation of SCLC with smoking mutational signature, characterized by high tumor mutational burden^3^. Though relative benefit was observed with immunotherapeutic agents like nivolumab and ipilimumab on comparison with chemotherapy in randomized clinical trials, studies have also shown that these benefits are limited to a small portion of patients; ideating that SCLC may be associated with a different immunological microenvironment^4^. Evaluation of biomarkers, such as smoking status, tumor mutation burden (TMB), and programmed cell death-ligand 1 (PD-L1) expression, have till date not been able to predict outcome^5^. Currently, SCLC is no longer considered a single-disease entity, with subtypes being classified into neuroendocrine (NE)-high and NE-low tumors, defined by distinct RNA gene expression profiles, which can possibly translate into different immunogenic profiles^6^. NE-high tumors, characterized by decreased immune cell infiltration are defined as cold or ‘immune desert’ phenotype, based on low levels of immune cell-related RNA expression while NE-low tumors are associated with increased immunogenicity, classified as ‘hot’ or ‘immune oasis’ phenotype, possibly more likely to respond to immunotherapies^7^. As most SCLCs are inoperable at the time of diagnosis, and relapses are frequently witnessed within the first year of treatment, use of newer modalities like immunotherapy appears cogent. The recent NCCN guidelines have also incorporated immunotherapy in ES-SCLC and SCLC with recurrence within six months of therapy^8^. However, in practical scenario owing to small improvement in overall survival and limited benefit extended to small number of patients, there arises an overwhelming need to identify biomarkers able to predict response to immune checkpoint inhibitors (ICPI) in SCLC patients.

Indoleamine-2,3-dioxygenase 1 (IDO1), which is a cytosolic enzyme catalyzing the rate-limiting step of tryptophan (Trp) catabolism to kynurenine (Kyn), has been found to be a key factor in defining cancer immunogenicity. Accumulation of Trp metabolites promotes the differentiation of T-reg cells and induces apoptosis of effector T-cells with consequent immunosuppression. IDO1 overexpression in tumors exploits immunosuppressive mechanisms to promote their spread and render poor prognosis. Thereby, serum Kyn/Trp concentration ratio has been explored as a potential biomarker of cancer-associated immune suppression in many tumors, including lung carcinomas, particularly non-small cell lung carcinomas (NSCLC)^9^. CTLA-4 (Cytotoxic T-lymphocyte-associated antigen-4, CD152), a CD28 homologue is translocated from intracellular storage to plasma membrane of T-cells, competitively binding to B7 ligands on antigen presenting cells (APCs) with higher affinity, thereby preventing CD28-mediated T-cell activation. However, soluble cytotoxic T-lymphocyte antigen 4 (sCTLA-4), one of the isoforms of CTLA-4, plays an important role in down-regulating the negative signal of CTLA-4 in T-cell responses and studies have suggested the favourable prognostic value of serum sCTLA-4 level in tumor patients^10^. Hence, along with expression of programmed death-1 ligand (PD-L1) on tumor cells, expression of CTLA-4 in the tumor microenvironment and its correlation with serum sCTLA- holds immense therapeutic potential.

Interferon-gamma (IFN-γ), a functionally pleiotropic cytokine modulating the expression of numerous proteins mainly upregulates PD-L1 expression^11^. IFN-γ expression in tumour tissues has been associated with response to treatment and studies have shown direct correlation of IFN-γ levels with response to ICPI in NSCLCs^12^. Levels of interleukin-2 (IL-2), a cytokine that binds to specific receptors expressed on T-cells and natural killer cells and thereby mediates their immune-stimulating effects, have been seen to correlate with long term survival in SCLC patients^13^. The therapeutic value of IL-2, highlighting its requirement in the efficacy of PD-1/L1 ICPI therapy has also been studied in NSCLC patients^14^. Tumor necrosis factor-alpha (TNF-α), an inflammatory cytokine promotes tumor growth and higher serum levels of TNF-α have been reported to be associated with poor prognosis in cancer patients. Preclinical TNF-α blockade has been touted to improve therapeutic effectiveness of ICPIs^15^. Although the prospective utility of all the above biomarkers in relation to ICPIs have been extensively studied and established in NSCLCs, their expression and future therapeutic potential in SCLCs remains largely undetermined.

In this study, we tried to characterize the immune landscape of SCLC by analysing the expression of various immune regulatory molecules on tumor cells as well as on immune cells present in the tumor microenvironment. Additionally, an attempt was also made to evaluate the levels of immune-regulatory, inflammatory and other protein mediators in blood of these patients, as biomarkers predicting potential response to ICPIs.

## Materials and methods

The study was of prospective design and approved by the Institute Ethics Committee of All Institute of Medical Sciences (AIIMS, New Delhi) [IEC-552/2016, RP-20/2016, OP-1/2021]. Cases of SCLC over a period of 3 years (2019–2022) were enlisted in the study. Histologically proven biopsy samples with adequate tissue in the formalin fixed paraffin embedded (FFPE) block were included. Paired peripheral blood samples were collected in all available patients. Respective serum and plasma of these cases were stored at −80 °C for further quantification studies. The study was performed in accordance with relevant guidelines/regulations (in accordance with the Declaration of Helsinki). Informed consent was obtained from all participants in the study, including patients and healthy controls.

**Figure 1:**
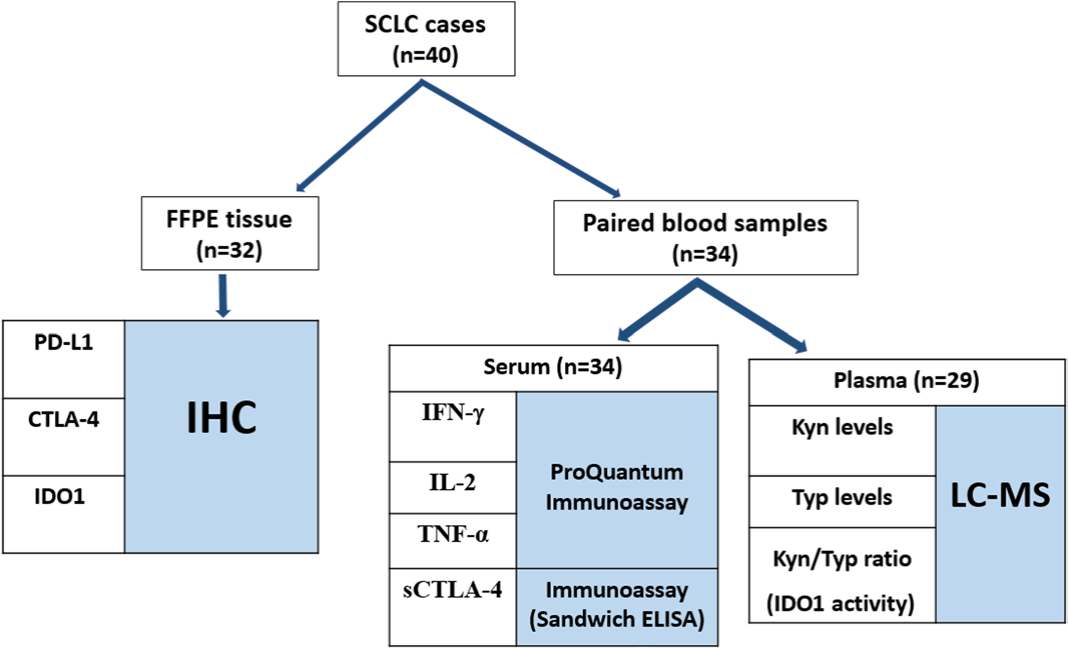
Workflow of the study along with available samples for analyses.

Immunohistochemistry was performed on FFPE samples for checking expression of inhibitory receptors (CTLA-4, PD-L1 and IDO1) and scoring was done on basis of the intensity and percentage of tumor cells stained. Matched serum samples collected from SCLC patients were quantified for following markers (IFN-γ, IL-2 and TNF-α) using immunoassay and additionally, for soluble CTLA-4 (sCTLA-4) using enzyme linked immunosorbent assay (ELISA). For comparison of above four mentioned serum markers, serum samples from 30 healthy individuals matched for age and gender were collected from donors in blood bank after ethical approval and informed consent, and processed in a similar manner. For determining plasma IDO1 activity, available plasma samples from SCLC patients were assessed for Kyn/Trp ratio, using liquid chromatography mass spectroscopy (LC–MS/MS) method. 40 plasma samples of age and gender-matched healthy controls were also analysed for comparison (collected after ethical approval and informed consent). A brief workflow of the study along with available samples for analyses has been shown in Fig. 1.

### Immunohistochemical (IHC) analysis of PD-L1, CTLA-4 and IDO1 in SCLC biopsies

5 micron sections from selected FFPE blocks were subjected to IHC for PD-L1 (clone SP263) which was performed on Ventana benchmark GX platform optimized with the Optiview DAB IHC detection kit. Sections of placenta were included as positive controls for endogenous PD-L1 expression. The guidelines for interpretation of PD-L1 IHC were adopted from the interpretation guide by Ventana Inc. for PD-L1 (SP263) assay staining of NSCLC. Any amount of membranous staining (whether discontinuous, circumferential or baso-lateral) of tumor cells at any intensity (greater than background staining) was considered positive while any cytoplasmic staining was disregarded. Tumor proportion score (TPS) was determined by assessing percentage of cells stained amongst viable tumor cells and 1% positivity was considered as the cut-off for PD-L1 positivity. Immunopositivity in the background immune cells like lymphocytes and intra-alveolar macrophages were not included in the scoring criteria. Other IHC markers such as CTLA-4 (sc-376016 clone, 1:50 dilution, Santa cruz) and IDO1 (VIN3IDO clone, 1:2000 dilution, eBiosciences) were performed manually. Sections from lymph nodes were used as controls. Immunohistochemical expression were scored based on the percentage of immunolabelled cells as: 0%—score 0, 1% to 33%—score 1, 34% to 66%—score 2 and 67% to 100%—score 3. Intensity of immunoexpression were scored as: score 1—mild, score 2—moderate, score 3—strong staining.

### Quantification of IFN-γ, IL-2 and TNF-α in serum

34 matched serum samples collected from SCLC patients were quantified for following markers (IFN-γ, IL-2 and TNF-α) using ProQuantum immunoassay kits (Thermofisher Scientific). ProQuantum high sensitivity immunoassays utilize a matched pair of target-specific antibodies, each conjugated to a DNA oligonucleotide. During antibody analyte binding, the two DNA oligos are brought into close proximity, which allows for ligation of the two strands and subsequent creation of a template strand for amplification. Serum samples were added to an antibody-oligo conjugate mix provided in respective kits and incubated at room temperature for 1 h or overnight at 4 °C. Following the initial incubation, master mix and ligase provided in the kit was added to the PCR assay plate followed by the instructed PCR thermal cycling: 20 °C hold for 25 min, 95 °C hold for 2 min, 40 cycles of 95 °C for 15 s and 60 °C for 1 min. After the run was complete, the results of qPCR file were imported using available ProQuantum cloud-based software. The data was then analysed to obtain protein concentration values in pg/mL.

### Quantification of sCTLA-4 in serum using ELISA immunoassay

The Human CTLA-4 solid-phase sandwich ELISA kit (enzyme-linked immunosorbent assay) was used to measure the amount of sCTLA-4 in 34 matched serum samples collected from SCLC patients. Appropriate standards diluted as per manufacturer’s instructions were added to the coated micro-well plate followed by samples from SCLC patients and healthy controls in 1:2 dilution and allowed to bind to the immobilized (capture) antibody at 37 °C for 2 h. The sandwich formed by the addition of the second (detector) antibody was then allowed to incubate overnight at 4 °C. Finally, substrate solution was added that reacted with the enzyme-antibody-target complex to produce measurable signals (absorbance at 450 nm). The intensity of these signals were then measured which was directly proportional to the concentration of sCTLA-4 present in the original sample.

### Assessment of IDO1 activity (Kyn/Trp ratio) in plasma by liquid chromatography mass spectrometry (LC–MS)

A previously published LC–MS/MS method with minor modifications was used for the analysis^16^.

#### Instrumentation

A triple quadrupole tandem mass spectrometer (4000 Q-Trap, AB Sciex, CA, USA) coupled with high performance liquid chromatography system (HPLC, Agilent Technologies, 1260 Infinity, CA, USA) was used. The HPLC system consisted of online degasser, quaternary pump, multi-sampler, thermostatted column compartment and variable wavelength UV detector. All the parameters of tandem mass spectrometer and HPLC were controlled by Analyst software, version 1.7.1 (AB Sciex, CA, USA) and Open-LAB control panel software (Agilent Technologies, 1260 Infinity, CA, USA), respectively.

### Chromatographic and mass spectrometer conditions

The chromatographic separation was achieved using Zic®-cHILIC column (100 × 4.6 mm, 3 µm, Merck, Germany). An isocratic mobile phase using acetonitrile with 0.1% formic acid (solvent A) and 5 mM ammonium acetate with 0.1% formic acid (solvent B) at a ratio of 60:40 (A:B) was used. The mobile phase was pumped at a flow rate of 0.5 mL/min with a total run time of 5.5 min. The column oven compartment was kept at ambient and the autosampler tray was maintained at 10 ± 1 °C. The standards and samples were loaded onto 96 well plate and injected at a volume of 2 µL.

All the analytes were ionized using Turbo Ion Spray (ESI) source operated at positive ion mode. Compound related parameters for Kyn, Trp and homatropine (HA) were optimized by infusing 100 ng/ml solution at 0.01 mL/min flow rate through Harvard pump (Harvard Company, Reno, Nevada, USA). HA served as an internal standard (IS) for Kyn and Trp. Multiple reaction monitoring (MRM) was used for the quantification of the analytes. Source dependent parameters like collision associated dissociation (CAD), curtain gas (CUR), ionizing voltage (iV), temperature, gas 1 and gas 2 were kept at 12 psi, 30 psi, 5500 V, 500 °C and 50 psi each, respectively.

### Calibration standard preparation

Kyn and Trp were accurately weighed at an amount of 10 mg and transferred to 10 mL amber colored volumetric flask. The powder was dissolved in 1:1 acetonitrile:water (10 mL). The stock solution was stored at 4 °C and used for further analysis. A calibration dilution was prepared in charcoal stripped human plasma where the range for Kyn was from 0.156 µg/mL to 5 µg/mL whereas Trp ranged from 0.625 to 20 µg/mL. 20 µL of each calibration standard was taken into a fresh tube and 200 µL of extraction solvent (80% acetonitrile in water with 0.1% formic acid and 50 ng/mL of HA as internal standard) was added to it. The solution was vortexed for 1 min and centrifuged at 7840*g* for 10 min at 4 °C. The supernatant was transferred to 96 well plate and subjected to LC–MS/MS analysis.

### Sample preparation

Plasma from patients as well as healthy controls were thawed at room temperature and vortexed briefly. As described above for the calibration standards, the samples were prepared in a similar manner.

### Statistical analysis

Statistical analysis was performed using the statistical software STATA Version 16.1 (STATA Corporation, College Station, TX, USA). Data were expressed as mean ± standard deviation (SD) or median [interquartile range (IQR)] for continuous variables and number (percentage) for categorical data, as appropriate. Shapiro Wilks test was applied to test the normality of distribution. Differences between the control and SCLC group were tested by using Independent t-test or Mann–Whitney test (for continuous variables) or Chi-square/ Fisher’s exact test (for categorical variables). The receiver operating characteristic (ROC) curve was applied to compare the strength of the classification of bio-markers between the control and SCLC group. A p-value less than 0.05 was considered statistically significant.

## Results

### Clinical demographics

A total of 40 SCLC cases were eligible to be included in this study. The age of the patients ranged from 27 to 76 years, with the median age being around 51.5 years. There was a male preponderance (male: female ratio being around 9:1). Smoking index (SI) ranged from 250 to 700 in 25 of 40 prospective cases, three patients exclusively chewed tobacco while data was not available in the rest of the cases. Data regarding tumor stage showed that 29 (72.5%) patients had ES-SCLC disease while 11 (27.5%) patients had LS-SCLC. The ECOG scores were 1, 2 and 3 in 8, 29 and 3 patients respectively; at the time of initial evaluation. 34 patients had progressive disease on latest follow-up visit including three patients who died of disease while 3 patients were lost to follow up (Table 1).

**Table 1:** Details of clinical characteristics of SCLC patients.

Parameter	Number of patients (n)	
Total number of patients	40	
Age range (median—51.5 years)		
< 25 years	0	0%
25–50 years	12	30%
51–75 years	27	67.5%
> 75 years	1	2.5%
Male:female ratio	36 males:4 females	9:1
Smoking index (SI) (available in 25 of 40 cases)		
< 250	2	8%
250–500	10	40%
501–700	13	52%
> 700	0	0%
History of tobacco chewing	3	7.5%
Associated co-morbidities	1 patient had diabetes mellitus	2.5%
ES-SCLC disease	29	72.5%
LS-SCLC disease	11	27.5%
ECOG scores at evaluation		
Score 1	8	20%
Score 2	29	72.5%
Score 3	3	7.5%
Follow up		
DOD	3	7.5%
Progressive disease	34	85%
Lost to follow up	3	7.5%

*DOD* died of disease, *ECOG* Eastern Co-operative Oncology Group-performance status scale, *ES-SCLC *extensive stage small cell lung cancer, *LS-SCLC *limited stage small cell lung cancer.

### Immunohistochemical (IHC) analysis of PD-L1, CTLA-4 and IDO1 in SCLC biopsies

**Figure 2:**
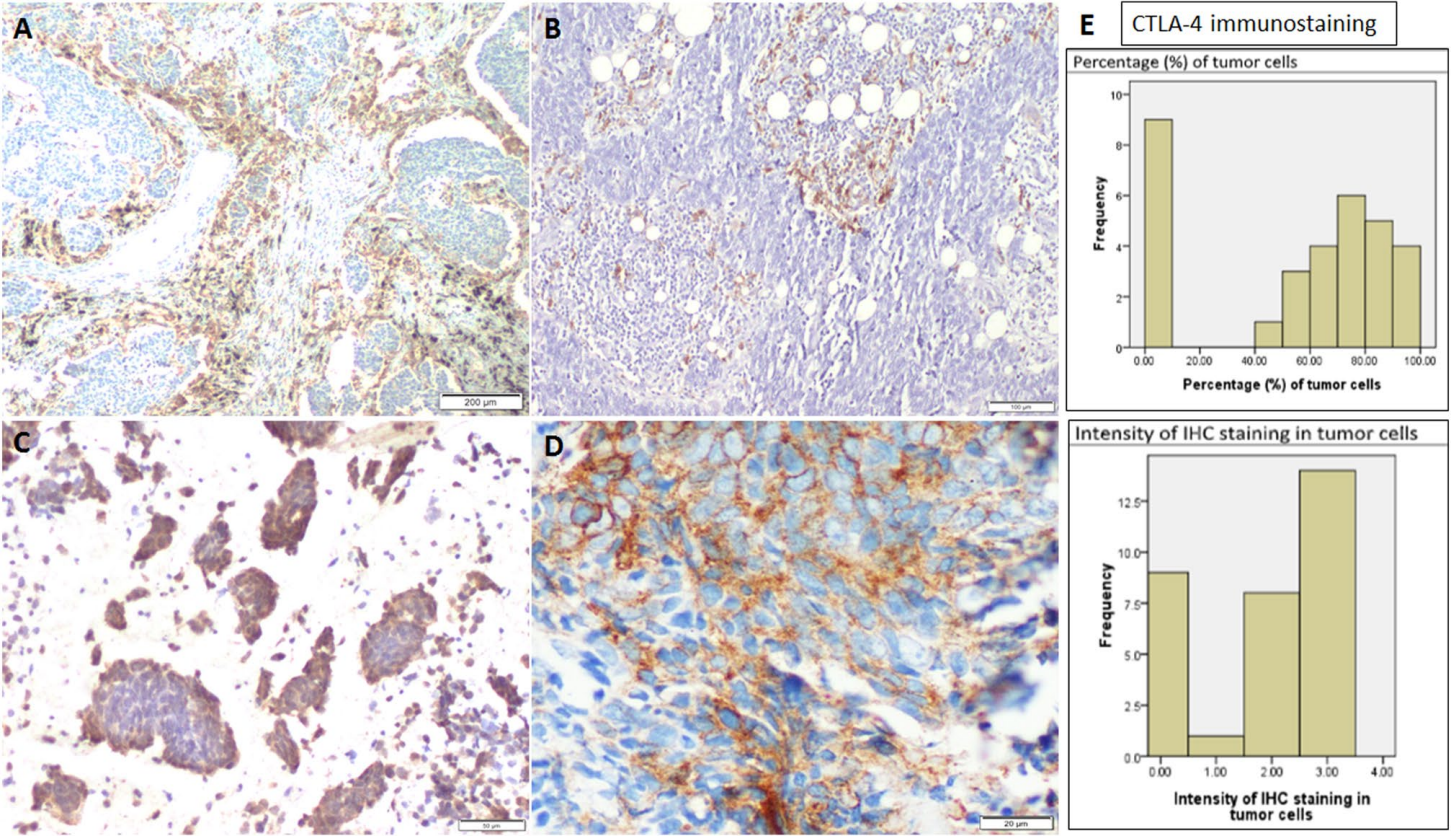
Immunohistochemical analysis of PD-L1, CTLA-4 and IDO1 in SCLC biopsies. (**A,B**) Immunostaining for IDO1 is seen in endothelial cells, intra-tumoral immune cells and stromal cells. (**C**) Immunostaining for CTLA-4 is seen in cytoplasm of tumor cells. (**D**) Strong membranous immunopositivity for PD-L1 is seen only in the tumor cells (TPS > 50%). (**E**) Histogram showing the intensity and percentage distribution of CTLA-4 immunoexpression in tumor cells amongst the SCLC cohort.

Only three cases (9.3%, 3/32) showed immunopositivity for PD-L1 in the tumor cells. The TPS in these three positive cases were 1%, 10% and > 50% respectively. Out of 32 cases, IDO1 was found to be positive in only two cases (6.2%). Strong IDO1 immunostaining (score = 3) were seen on approximately 75% and 80% of endothelial cells, intra-tumoral immune cells and peritumoral stromal cells in these two cases respectively. Immunostaining for CTLA-4 was seen in cytoplasm of tumor cells. CTLA-4 immunostaining was present in majority of the cases (71.8%, 23/32). The graphical representation of the intensity and percentage distribution of CTLA-4 immunoexpression across the SCLC cohort have been included in Fig. 2.

IHC analyses could not be performed on 8 cases due to exhaustion of representative tumor tissue in the FFPE block, after performing routine immunohistochemical testing at the time of diagnosis.

### Quantification of IFN-γ, IL-2 and TNF-α and sCTLA-4 in serum

**Figure 3:**
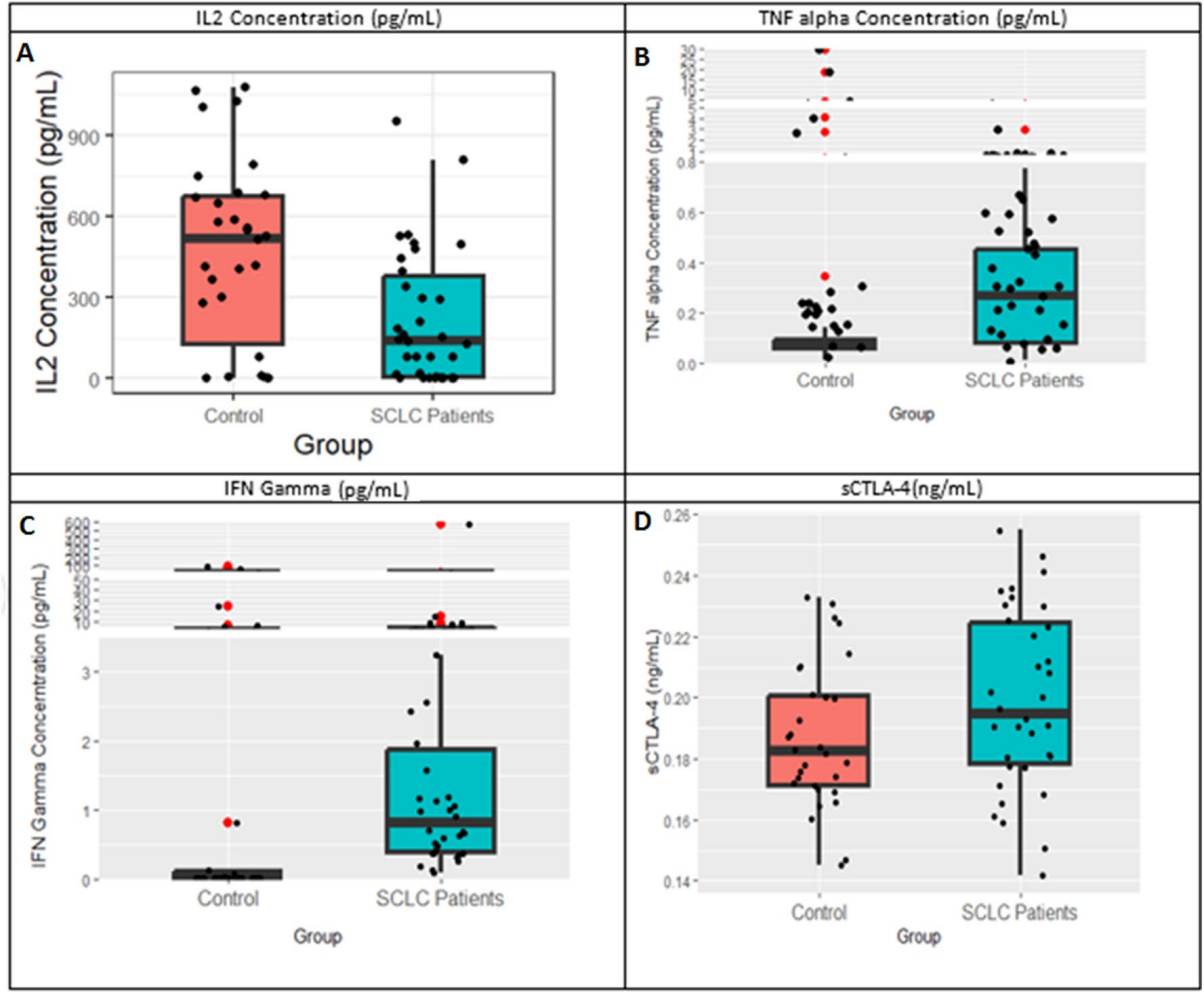
Quantitative estimation of serum cytokine levels and sCTLA-4. Box-whisker plots showing the distribution of serum levels of (**A**) IL-2, (**B**) TNF-α, (**C**) IFN-γ and (**D**) sCTLA-4 in SCLC patients and healthy controls.

For TNF-α, IL-2 and IFN-γ estimation, serum samples of 34 SCLC patients were quantified using ProQuantum immunoassay kit (done in triplicates). Serum concentrations of IFN-γ, IL-2 and TNF-α were interpolated against a standard curve. The normality assumption was violated among all these three biomarkers, the box-whisker plots showing the distribution are presented in Fig. 3. The concentration of IFN-γ was found to be significantly higher in SCLC patients [median (IQR) = 0.813 {0.369 to 2.088} pg/mL; range = 0.099 pg/mL to 576.4 pg/mL; p-value < 0.001] than healthy controls [median = 0.0165 (0.013 to 0.295) pg/mL]. The IL-2 concentrations were found to be significantly lower in SCLC [median (IQR) = 140.43 {3.899 to 407.44} pg/mL; p-value = 0.003] than in healthy controls [median = 519.8 (60.66 to 681.28) pg/mL]. The TNF-α concentration of total 34 samples interpolated against a standard curve showed that concentration in SCLC patients was significantly higher [median (IQR) = 0.269 {0.066 to 0.460} pg/mL; p-value = 0.025) than healthy controls [median (IQR) = 0.0695 {0.054 to 0.107} pg/mL], with exception of two controls who showed high levels of TNF-α (Fig. 3).

For sCTLA-4 estimation, serum samples of 34 SCLC patients were quantified using ELISA immunoassay (done in duplicates). The sCTLA-4 concentration of total of 34 samples was interpolated against a standard curve and concentrations were found to be normally distributed. The mean of sCTLA-4 was slightly higher in SCLC patients [mean (SD) = 0.199 (0.030) ng/mL; range = 0.142 ng/mL to 0.255 ng/mL] than healthy controls [mean (SD) = 0.182 (0.024) ng/mL] but not statistically significant (p-value = 0.08) (Fig. 3).

**Figure 4:**
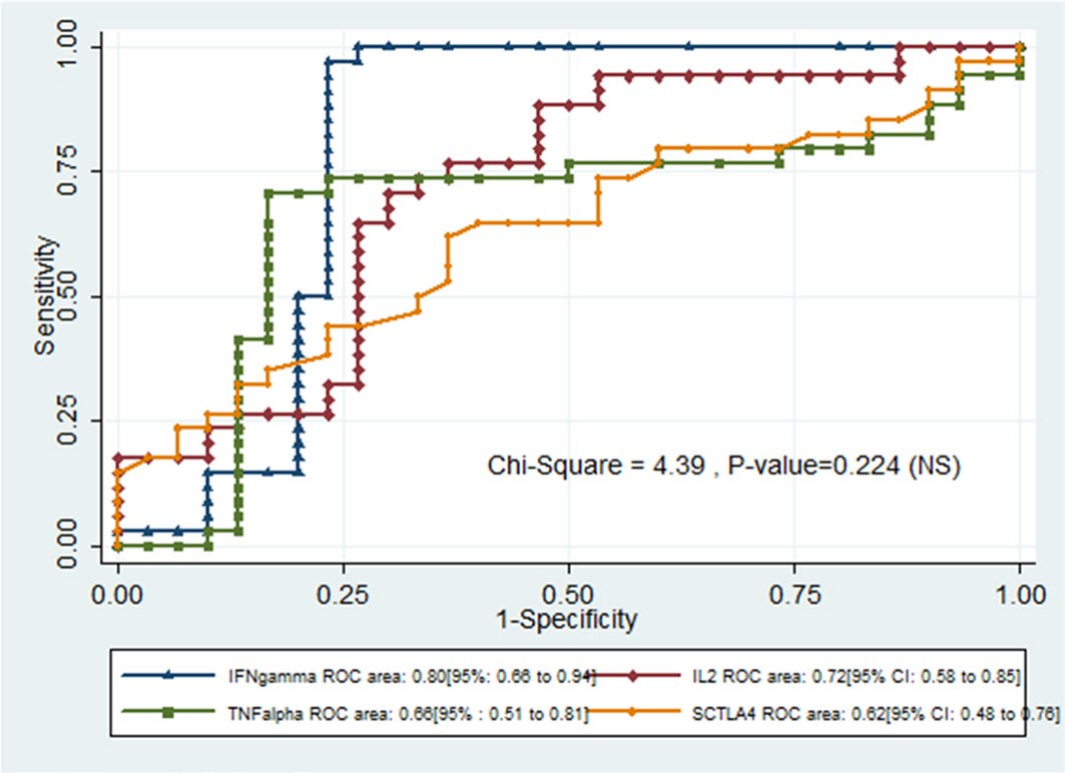
ROC curve comparing the discrimination potential amongst the serum biomarkers.

The comparison of the area under the ROC curve revealed no significant difference between the discrimination potential of these bio-markers (p-value = 0.224). The respective areas of IFN-γ, IL-2 and TNF-α and sCTLA-4 under the ROC curve and their corresponding 95% confidence interval are presented in Fig. 4.

Quantification could not be carried out in 6 serum samples as they were technically unsuitable.

### Assessment of IDO1 activity (Kyn/Trp ratio) in plasma by LC–MS

**Figure 5:**
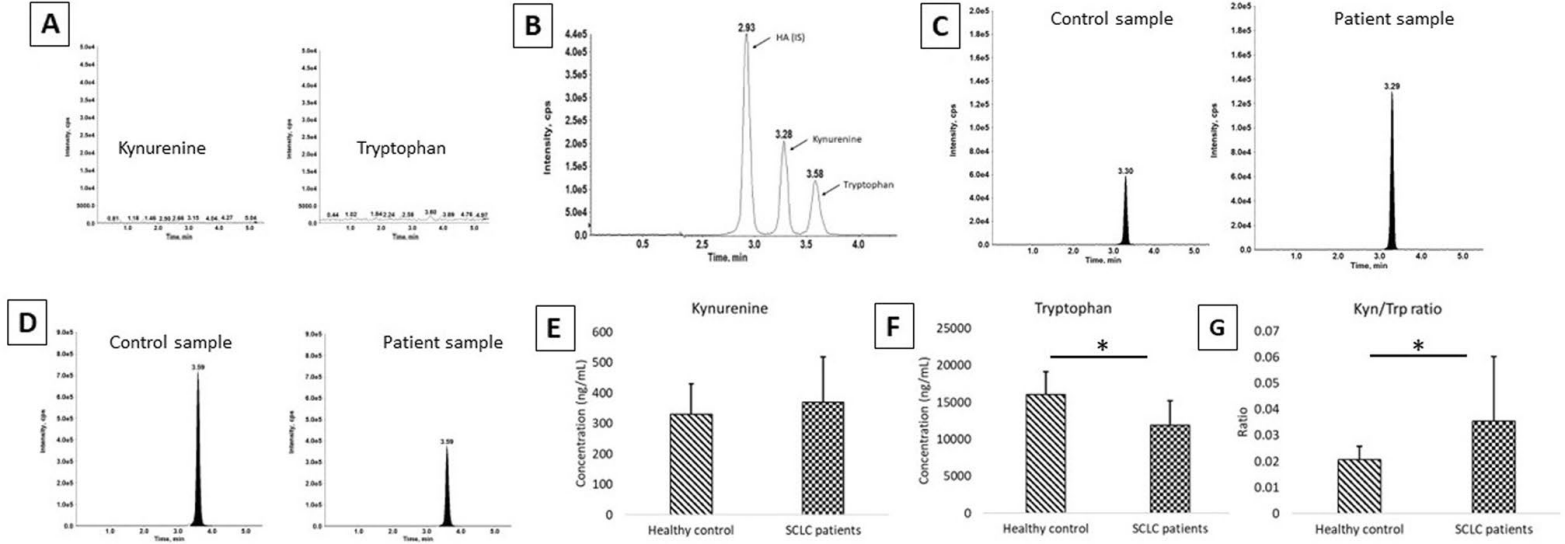
Quantification of kynurenine and tryptophan in healthy control and SCLC patients using LC–MS/MS. (**A**) Stripped plasma blank showing no peaks for endogenous Kyn and Trp. (**B**) Chromatogram showing separation of Kyn, Trp and homatropine (HA) which is used as an internal standard (IS). (**C**) Representative plasma concentration of Kyn and (**D**) Trp in control and patient sample, respectively. (**E**) Kyn levels in the plasma samples of healthy control and SCLC patients. (**F**) Trp levels in the plasma samples of healthy control and SCLC patients. (**G**) Kyn/Trp ratio of healthy control and SCLC patients.

The hydrophilic compounds Kyn and Trp were separated using hydrophilic interaction liquid chromatography (HILIC). Charcoal stripped plasma was used as the matrix for the preparation of calibration standards and it was free of endogenous Kyn and Trp. The Kyn levels in SCLC patients (n = 29) were found to be higher compared with healthy controls (n = 40). SCLC patients had lower levels of Trp when compared with healthy controls, the difference was also found to be statistically significant (p < 0.05). The assessment of IDO1 activity using Kyn/Trp ratio revealed 1.7 times higher values for SCLC patients compared with healthy control (Fig. 5) in a significant manner (p = 0.007).

## Discussion

In recent times, ICPIs have become the most widely sought after therapeutic regimen, employed alone or in conjunction with chemotherapy, owing to their effective use in many malignancies. Though multiple randomized clinical trials have demonstrated the efficacy of the combination of ICPIs like PD1/PD-L1 inhibitors and anti-CTLA4 monoclonal antibodies along with chemotherapy in the first-line treatment as well as maintenance therapy of ES-SCLC, their practical perquisite in SCLC still remains under speculation. The lack of biomarkers predicting response to therapy compounded by immune related adverse effects owing to unjustified usage of ICPIs acts as hindrances. In this study, we tried to analyse the expression of different biomarkers in SCLC so as to evaluate whether it is possible to dissect a sub-group among the SCLC cohort who can be predicted to be potential responders to ICPIs.

Kyn-pathway controlled IDO1 has been associated with poor prognosis in various cancers. Increased IDO1 levels, reflected by decreased Trp and elevated Kyn concentrations in the peripheral blood has been observed to be related to tumor progression and poor clinical outcome. IDO1 induction in malignant cells and their microenvironment has also been hypothesised as a key mechanism to modulate anti-tumor immune response, helping in escaping immune surveillance. Decreased Trp availability and accumulation of its metabolites, such as kyn also directly affects immune T-cell proliferation and functions, and induce their apoptosis. Based on these mechanisms, it can be speculated that elevated IDO1 levels can serve as a potential biomarker, highlighting decreased immune cell surveillance and tumor progression. IDO1 activity has been studied in NSCLC where it has been seen to be directly proportional to tumor stage and aggressiveness. Studies in human lung cancer cell lines demonstrated that though IDO mRNA can be constitutively expressed by lung cancer cells, higher IDO1 expression observed in patient samples could be attributed to production of the enzyme by other cells recruited in the tumor microenvironment and the peri‑tumoral area, thereby mediating its immune conditioning^17^. Studies trying to correlate IDO1 levels with response to ICPI regimens have found that in certain tumors like stage IV melanoma and renal cell cancer treated with anti-PD-1 therapy, increase in Kyn/Trp during therapy compared to baseline was seen to be associated with significantly reduced progression-free survival^18^. IDO1 immuno-expression has been observed not only in tumor cells but in the surrounding stroma, as well. Thereby IDO1 immunolabelling in tumors has been seen to show three distinct cellular expression patterns which includes expression in tumor cells, interstitial cells in lymphocyte-rich areas in the tumor stroma or endothelial cells^19^. These patterns can be seen individually or in combination. In our study, the levels of kyn/try were assessed using hydrophilic interaction chromatography (HILIC), which offers better separation of hydrophilic analytes^20^. Depleted tryptophan levels and increased levels of kynurenine, indicating raised IDO1 activity were seen in our SCLC cohort as compared to healthy controls and the values were also statistically significant. However, on evaluating IDO1 immunohistochemical expression, we could elicit immunopositivity in only two cases. Nevertheless, a plausible explanation for this contrast in expression, could be due to defective expression of protein at the tissue level.

IDO1 expression is interconnected with other immune checkpoint molecules like CTLA-4. In the local tumor microenvironment, CTLA-4 expression has been seen to upregulate IDO1, which reciprocally promotes T-reg activation. This interplay can also be evinced in peripheral blood, where IDO1 expression has been seen to be associated with increased CTLA-4 + Tregs.

Soluble CTLA-4 (sCTLA-4), produced by alternatively spliced messenger RNA (mRNA) has functions which are contrary to the classical immunosuppressive effect of CTLA-4. Studies have demonstrated that sCTLA-4 functions in down-regulating the action of CTLA-4 in T-cell responses as sCTLA-4 interferes with CD80/CD86:CTLA-4 interactions, thereby blocking the negative signal imparted via CTLA-4 in the later phases of T-cell response. sCTLA-4 in pleural effusions of mesothelioma patients has even been shown to behave as a statistically significant positive prognostic factor^10^. Though sCTLA-4 concentration was higher than controls in our SCLC patients, it was not statistically significant. On the other hand, 71.8% of our cases showed CTLA-4 immunohistochemical staining along with significantly raised IDO1 activity. These findings highlighted probable similar interactions between CTLA-4, s-CTLA-4 and IDO-1 as described in studies above.

Interferons are potent IDO1 inducers and part of the IDO1 expression by tumor cells might be attributed to IFN-γ. As tumor-infiltrating lymphocytes (TILs) are a predominant source of IFN-γ, they might upregulate IDO1, thereby potentially contributing to tumor immune escape^12^. IDO1 induction can also be potentiated by other pro-inflammatory cytokines, such as TNF-α. Though studies in NSCLC have highlighted that TNF-α signalling is involved in lung tumor progression and response to immunotherapy, the exact mechanisms modulating it remains to be unearthed^15^. The negative role played by TNF-α secreted by tumor associated macrophages, in promoting cell glycolysis, tumor hypoxia and decreased PD-L1 expression has been highlighted in few studies; while others have observed increased serological levels of TNF-α to be associated with improved response to anti-PD-1 treatment and survival^15^. These observations make the role of TNF-α seem rather cryptic and like a double edged sword. Tryptophan-catabolizing enzyme IDO1 also results in infiltration by FoxP3 + Tregs, thereby mediating suppression of effector T-cell function and causing T cell-intrinsic anergy. This is characterized by production and proliferation of defective IL-2^14^. Fischer et al. in their study on SCLC patients found that IL-2 levels served as independent prognostic factors and high serum levels at baseline directly correlated with long term survival^13^. In our study, IL-2 levels were found to be significantly lower as compared to controls (p = 0.003). In addition, serum IFN-γ (p-value < 0.001) and TNF-α levels (p-value = 0.025) were significantly higher in the SCLC cohort compared to healthy controls; suggesting marked immune suppression.

IDO1 induced T-regs are seen to reciprocally upregulate PD-L1 and PD-L2 expression on target dendritic cells^17^. Studies analysing PD-L1 positivity in neuroendocrine carcinomas including SCLC have cited variable percentages ranging from 5.8% to as high as 71.6%^5^. However, this conflicting results could be attributed to use of unconventional clones of PD-L1 for analysing IHC expression and different methodologies adopted for calculation of scores, which includes counting immune-expression in immune cells in addition to tumor. Studies using FDA approved clones and cut-offs for estimating PD-L1 positivity have usually found low rates of PD-L1 immunopositivity in SCLC^5^. In our study, we could elicit PD-L1 immunopositivity in tumor cells in only three cases with TPS scores of 1%, 10% and > 50%. However, we noticed immunopositivity in the immune cells in the tumor stroma in approximately one-fourth (25%) of our cases. In the absence of well-defined criteria to assess PD-L1 status on immune cells alone, using FDA approved clones; whether to interpret this finding as a hint towards immune privilege nature remains a bone of contention.

To summarise, SCLC patients show immune suppressive milieu in their peripheral circulation and can be potentially benefitted by appropriate immuno-modulatory regimen. Owing to various clinical trials advocating the use of anti-PD-L1 drugs in recurrent or ES-SCLC quoting significant difference in survival rates, the need for various predictive biomarkers arises, which might help in decision making regarding sustaining or quitting ICPI regimens. Theragnostic biomarkers like IDO1, s-CTLA-4, IFN-γ, TNF-α and IL-2 have been studied in various tumors, and seems to hold significant potential in mapping the tumor microenvironment and predicting response to ICPIs. However apart from IL-2, till date, there has been hardly any studies exploring the potential of using these biomarkers in SCLC. The findings in our study suggest that these biomarkers are expressed in SCLCs also and might act in a similar manner in predicting prognosis and response to different therapeutic regimens as has been established in other tumors, including NSCLC. However, our study cohort was small in size and more studies incorporating larger groups are warranted to validate whether our findings can be extrapolated to SCLCs per se. Another limitation in our study was that owing to aggressive nature of SCLC and difficulty in following up patients, we could not evaluate the fluctuation of these biomarkers in relation to response to different therapeutic regimens or carry out survival analyses. Howbeit, we proffer that evaluation of these biomarkers particularly IDO1 at baseline appears cogent not only as a prognostic marker but also hold potential as a therapeutic target. Based on IHC expression of immune co-inhibitory molecules in our cohort, tumor cells of SCLC were found to be poor immuno-expresser of PD-L1 and IDO1, while CTLA-4 immunolabelling was seen in a fair number of cases. In the absence of PD-L1, CTLA-4 immuno-expression in tumor cells and related adjuncts like s-CTLA-4 levels in serum may be studied so as to analyse whether they conform as biomarker for predicting responsiveness to ICPIs.

## Data Availability

The datasets generated during and/or analysed during the current study are available from the corresponding author on reasonable request.
